# Vocalizations during post-conflict affiliations from victims toward aggressors based on uncertainty in Japanese macaques

**DOI:** 10.1371/journal.pone.0178655

**Published:** 2017-05-30

**Authors:** Noriko Katsu, Kazunori Yamada, Masayuki Nakamichi

**Affiliations:** 1 Graduate School of Arts and Sciences, The University of Tokyo, Tokyo, Japan; 2 Graduate School of Human Sciences, Osaka University, Suita, Osaka, Japan; Universita degli Studi di Pisa, ITALY

## Abstract

We investigated the use of vocalizations called “grunts,” “girneys,” and “coos” accompanied by post-conflict affiliative interaction between former opponents (reconciliation) in Japanese macaques (*Macaca fuscata*). Although reconciliation functions to repair bonds, such interactions sometimes entail risks of receiving further aggression. Vocalizations can be used at a distance from the former opponent; thus, we predict that vocalizations are used particularly by victims of a conflict, and are frequently used in situations of uncertainty when it is difficult for them to estimate whether the former opponent will resume aggression. In addition, we predict that vocalizations are effective in preventing further aggression. To test these hypotheses, we conducted observations of post-conflict and matched-control situations in female Japanese macaques living in a free-ranging group. We found that former opponents tended to be attracted to each other within the first minute following a conflict, thus demonstrating reconciliation behavior. Vocalizations were more frequently used by the victims in post-conflict interactions than under control situations; however, this tendency was not found in aggressors. When affiliation with the former opponent occurred, victims were more likely to use vocalizations towards less familiar opponents. These findings suggest that Japanese macaques used vocalizations more often when interacting with less predictable former opponents. Victims were more likely to receive aggression from former aggressors when engaged in affiliations with them than under no such affiliations. No significant differences were found in the probability of the victims receiving aggression, regardless of whether they used vocalizations; thus, whether the victim benefits from using vocalizations in these contexts remains unclear. Japanese macaques form despotic societies and therefore, further aggression was inevitable, to some degree, after a conflict. The use of vocalizations by a victim was found to depend on the nature of their relationship with the aggressor; however, the effectiveness of this behavior requires further investigation.

## Introduction

A post-conflict (PC) affiliative interaction between former opponents is generally labeled as reconciliation [1, 2]. Reconciliation often occurs between individuals who benefit from maintaining a stable relationship (the valuable relationship hypothesis [3–6]); thus, reconciliation restores damaged relationships and prevents future loss of benefits [6–9]. However, some studies have shown that reconciliation entails risks of receiving further aggression from former opponents [10]. The high probability of receiving further aggression is believed to lower the occurrence of reconciliations [11].

A previous research has indicated that forms of post-conflict interactions differ based on the nature of the relationship between opponents and the risk of renewed aggression. For instance, reconciliation among stump-tailed macaques (*Macaca arctoides*) is categorized into two general forms: grooming/contact sitting and socio-sexual behaviors (e.g., mounting) [12]. The former behaviors occur frequently when opponents are kin or friends, whereas the latter behaviors occur when opponents are non-kin or non-friends [12]. Male hamadryas baboons (*Papio hamadryas hamadryas*) who are non-friends often engage in non-contact greetings, such as approaches or facial expressions (e.g., lip-smacking and ear-flattening) after conflicts [13]. Mandrills (*Mandrillus sphinx*) direct facial expressions or postures toward former opponents who are likely to instigate renewed aggression [14]. These non-contact behaviors are effective as well as contact behaviors in reducing further aggression [14]. These studies suggest that forms of reconciliation directed at non-friends or dangerous individuals are different from those directed at friends, and that the purpose of these behaviors is aimed to reduce further aggression. These studies also highlight that the forms of reconciliation behaviors are often species-specific.

Macaques, including Japanese macaques (*Macaca fuscata*), use soft vocalizations known as grunts and girneys [15, 16], which are often used during face-to-face interactions [16]. In addition, Japanese macaques occasionally use a contact call, known as coo calls, in the same fashion as grunts and girneys; these vocalizations communicate non-hostile intent when targeted at a specific individual that is in close proximity [17, 18]. These three vocalizations are more or less equivalent to grunt vocalizations observed in baboons (*Papio* spp. [19, 20]), and indeed grunts are also used during reconciliation interactions in macaques as well as baboons [21–23]. A previous study on female baboons focused on the function of vocal behaviors after a conflict from a higher-ranking opponent [24]. This vocal behavior was intended to signal benign, non-hostile intent toward a subordinate opponent, and reduce uncertainty in the recipients about whether the conflict would continue and further aggression would occur [25].

Japanese macaques form despotic and nepotistic societies with steep dominance relationships and kin-biased social relationships [11, 26]. In this species, victims of a conflict are usually subordinates to aggressors, with former aggressors often engaging in post-conflict aggression toward their opponents [10]. Subordinate opponents are, therefore, expected to benefit from the use of vocalizations, which can be used at a distance from the aggressors to convey non-hostile intent. Victims may assess whether aggressors have a non-hostile attitude and whether post-conflict interaction with the former opponent will entail a risk of receiving further aggression [14]. However, it has not been investigated whether Japanese macaques who engage in conflict, especially subordinate counterparts, utilize vocalizations based on uncertainty about the behavior of the former opponent.

We aimed to clarify whether vocalizations are used on the basis of the level of uncertainty in post-conflict situations, and whether vocalizations play a specific role among Japanese macaques. We assume that uncertainty increases in post-conflict situations and during interactions with less-familiar or higher-ranking opponents. We first examined whether victims used vocalizations more frequently in post-conflict situations than in control situations. We then examined whether vocal behaviors occurred more frequently with less-familiar and higher-ranking individuals. Finally, we examined whether vocalizations function to prevent further aggression from the former opponent, that is, we examined whether the immediate risk of receiving aggression was lower following post-conflict interactions with vocalizations than those without vocalizations.

## Materials and methods

### Ethics statement

We conducted this study in accordance with the Regulations on Animal Experimentation at Osaka University. The study was approved by the Animal Research Committee of the Graduate School of Human Sciences at Osaka University in Japan (No. 21-14-10). As the study area was privately owned, we conducted observations with the permission of the manager of Iwatayama Monkey Park, Kyoto, Japan.

### Study site and subjects

The present study was conducted in a free-ranging group of Japanese macaques belonging to the Arashiyama group at the Iwatayama Monkey Park (35°00′N and 135°67′E), Kyoto, Japan. The Arashiyama group has been provisioned since 1954 [27], after which maternal kinship was recorded. Individual macaques were identified based on their natural facial and physical characteristics. Dominance relationships among adult females were identified by the first author (K.N.) based on dyadic aggressive interactions (see below) and supplanting (approach-retreat interactions). We calculated a linearity index *h'* [28] and the linearity of the dominance relationships using MatMan 1.1 (Nordus). The group was fed wheat grains and soybeans five times daily, four times on the provisioning ground and once near the sleeping site. We collected data for dominance relationships, PC or matched-control (MC), and familiarity (see below) between March 2013 and March 2015, a period that included both mating seasons (from March to July) and breeding seasons (from October to February). The Arashiyama group consisted of 126 individuals [88 adult (>4 y) females, 10 adult males, 10 immature females, and 18 immature males] at the beginning of the study, with 117 individuals (86 adult females, 10 adult males, 7 immature females, and 14 immature males) remaining at the end of the study.

### Data collection

Behavioral observations were conducted between 09:00 h and 17:30 h, when the monkeys remained near the provisioning area, based on the post-conflict-matched-control (PC-MC) method [8, 29]. We collected data solely pertaining to dyadic conflicts between adult females; when the observer detected an aggressive interaction (physical aggression: bite, grab, and slap; non-physical aggression: chase and threat), the identity of the monkeys involved and their role (aggressor or victim) were recorded. We also recorded the occurrence of counter aggression (i.e., aggression from victims). After the aggression ended, the observer began a 5-min focal observation of either the aggressor or victim (i.e., PC sessions). We alternated between observing the aggressor and the victim as the focal subject between PC sessions.

The following behaviors were video-recorded (SONY HDR-CX560V) by the observer using the all-occurrence sampling method in PC and MC sessions: occurrence of aggression received by the subject, the first affiliative behavior(s) between the subject and the former opponent in PC sessions, and between the subjects and any other individuals (including the former opponent) in MC sessions. Affiliative behaviors were defined as non-aggressive physical contact, including grooming (giving and receiving); approaches within arm’s length; and giving vocalizations such as grunts, girneys, and coos, which are distinct vocalizations and therefore distinguishable by ear [18]. If the subject emitted calls with their face oriented toward their former opponents within 5 m, they were classified as ‘the subjects gave vocalizations’. We postponed a PC session if aggression between the former opponents resumed within 30 s [8, 22]. MC sessions were observed for the same subject as observed in each PC session, at least one observation day later but within 2 weeks, at approximately the same time of day (±2 h), and were initiated when no social interactions (affiliative or aggressive) were observed for the subject in the previous 5 min. MC sessions were conducted using the same procedures used for the PC sessions; however, affiliative interactions recorded in MC sessions were not limited to those between both former opponents because MC sessions were initiated regardless of the proximity between former opponents. Observations were not conducted within 20 min after artificial feeding, as reconciliation was less likely to occur following aggression over food [30]. A total of 605 pairs of PC-MC sessions were collected, that focused on 91 adult females. We recorded 300 PC-MC sessions in which 65 females were the aggressors and 305 sessions in which 79 females were the victims. Each individual was observed as the focal subject from one to 19 times, with a median of five times.

In addition to PC-MC observations, we collected data on the baseline levels of affiliative interactions between adult females as an indicator of familiarity. We conducted line census observations, in which all visible adult females were recorded by an observer while walking along a predetermined route. We used this observation method instead of scan sampling because individuals were dispersed over a wide range and could not always be observed from a single location simultaneously. We recorded the activity of each adult female during: (1) physical contact including grooming, or within 3 m of another adult female, or (2) alone. Intervals between each census were at least 1 h in length, and a total of 172 census sessions were performed over 95 days.

### Data analysis

#### Index of familiarity and dominance relationship

We calculated the index of familiarity between two adult females (A and B) by dividing the number of sessions that A had contact with or proximity to B by the number of sessions A was recorded in the line census. The familiarity index of A with B and that of B with A is not necessarily identical, given that the relative familiarity may not be equal for each individual (3). Because the level of familiarity between two individuals may change over time, we calculated the familiarity index separately into two evenly divided observation periods: (1) from March 2013 to March 2014 and (2) from April 2014 to March 2015. The former and latter periods included 87 and 85 census sessions, respectively. Each focal subject was recorded in 12–55 (median: 29) sessions in the former period and in 10–45 (median: 23) sessions in the latter period. The familiarity indices ranged from 0 to 0.73 (median: 0). The correlation between the familiarity indices of the former and latter period was significant, but relatively low (Pearson’s product-moment correlation: N = 155, r = 0.182, P = 0.023). This is probably because presence or absence of an infant has an impact on social interaction between females in Old World monkeys [31, 32].

Rank differences between subjects and former opponents were calculated by subtracting the absolute dominance rank of the former opponent from that of the subject. Positive values for rank differences indicated that the subject was subordinate to the former opponent, and negative values indicated that the subject was dominant. Values for the rank differences ranged from –89 to 89.

#### Statistics

We conducted all analyses in R version 3.2.0 [33]. We first tested for the presence of reconciliation on a minute-by-minute basis by dividing each PC and MC session into five one-minute timeframes, and comparing the timing of the affiliative behaviors between the timeframes of the PC and the corresponding MC sessions, based on the PC-MC method [29]. We included affiliative behaviors with both the former opponent and other individuals in the analysis of the MC. Subjects that were observed fewer than three times were excluded; as such, 76 subjects were included in the analysis. We categorized the PC-MC pair as ‘attracted’ if affiliative behaviors occurred only in or earlier in the PC than in the MC timeframes. The pair was categorized as ‘dispersed’ if they occurred only in or earlier in the MC than in the PC timeframes. The pair was categorized as ‘neutral’ if the behaviors occurred at the same time in the PC and the MC timeframes, or if they did not occur in neither the PC or the MC timeframes. We compared the proportion of attracted PC-MC pairs with the proportion of dispersed pairs for each subject from the first to the fifth timeframe using Wilcoxon signed-rank exact tests with the exactRankTests package in R [34].

In the following analyses, we conducted generalized linear mixed models (GLMMs) with a binomial distribution (logit link) using the lme4 package in R [35]. GLMMs allowed the use of multiple sampling from the same subjects by controlling the non-independence of data by a random effect. GLMMs were conducted separately depending on the role of the subjects as either aggressors or victims because the factors affecting the occurrence of affiliation and other behaviors were assumed to be different from aggressors and victims. We set the ID of the subject and the former opponent as random effects. Random slopes for all fixed effects were included where applicable. We first compared the full and the null models (model including only random effects) using likelihood ratio tests [36] and confirmed that the overall model was significant. When the models included the effect of multilevel (e.g., type of affiliative behaviors), we investigated the significance of that effect using the likelihood ratio test comparing the full model with a model that did not include that effect. The response and explanatory variables of each GLMM are summarized in Table 1. All tests were two-tailed with significance levels set at *P* < 0.05.

**Table 1:** Definition for response and explanatory variables used in GLMMs.

Analyses ID	Variables		Definition	Type
	Response variables			
1		Occurrence of affiliative interaction in PC	Whether affiliative interaction with the former opponent occurred in PC.	Binary (Yes/No)
2		Risk of receiving aggression in PC and MC	Whether a subject received aggression from other individuals in PC and MC.	Binary (Yes/No)
3		Risk of receiving aggression in PC	Whether a subject received aggression from the former opponent in PC.	Binary (Yes/No)
4		Vocal use in PC and MC	Whether a subject gave vocalizations toward an interacting partner during an affiliative interaction in PC and MC.	Binary (Yes/No)
5		Vocal use in PC	Whether a subject gave vocalizations toward the former opponent during an affiliative interaction in PC.	Binary (Yes/No)
6, 6'		Consequences of vocalizations	Whether the subject received aggression from the former opponent in PC.	Binary (Yes/No)
	Explanatory variables			
1, 3, 5, 6, 6'		Rank difference	Rank difference between a subject and the former opponent. Positive value indicated that the subject was subordinate to the former opponent	Continuous
1, 3, 5, 6, 6'		Familiarity	Familiarity index between a subject and the former opponent. High value indicated that the subject was more familiar with the former opponent.	Continuous
1, 3, 5, 6, 6'		Physical aggression	Whether a conflict included physical aggression.	Binary (Yes/No)
1, 3, 5		Counter-aggression	Whether a conflict included counter aggression.	Binary (Yes/No)
2, 4		PC or MC	Whether a session was PC or MC.	Binary (PC/MC)
3		Affiliation with the former opponent	Whether affiliative interactions with the former opponent occurred.	Binary (Yes/No)
6		Victim used vocalization	Whether a subject gave vocalizations toward the former opponent during an affiliative interaction.	Binary (Yes/No)
6'		Type of behavior	Whether an affiliative interaction consisted of vocalizations with physical contact or approach, vocalizations only, physical contact or approach only.	Categorical (vocalizations with physical contact or approach/ vocalizations only/physical contact or approach only)

#### Factors affecting affiliative interactions between the former opponents

We investigated the factors that influenced the occurrence of affiliative interactions between former opponents for all PC sessions (Model 1; Table 1). The response variable was whether affiliative interactions occurred in the PC session. The fixed effects consisted of the degree of familiarity and rank differences between former opponents, whether the original conflict included physical aggression, and the occurrence of counter-aggression.

#### Risk of receiving aggression

We investigated the probability of receiving aggression between all PC and MC sessions (Model 2; Table 1). The response variable of this GLMM was whether a subject received aggression from other individuals, and the explanatory variable was whether the session was PC or MC. After confirming an increase in the risk of aggression in PC, we compared the probability of receiving aggression based on whether aggressors and victims had affiliative interaction with their former opponent following the conflict (Model 3; Table 1). We conducted a GLMM on all PC sessions, with the response variable consisting of whether subjects received aggression from the former opponent. If affiliative behaviors with the former opponent occurred, we counted aggression from the initiation of the affiliative behaviors, whereas if no affiliative behaviors occurred, we counted aggression for the entire PC session, excluding aggression that occurred within the first minute. The fixed effects consisted of whether affiliative behaviors occurred with the former opponent in each PC session, the degree of familiarity, the rank differences between former opponents, whether the original conflict included physical aggression, and the occurrence of counter-aggression.

#### Vocal use after conflict

We investigated whether vocal use increased after a conflict in PC and MC sessions in which affiliative interactions (vocalizations, contact, grooming, or approach) occurred (Model 4; Table 1). The response variable was whether the subject gave vocalizations during contact, grooming, and/or approach behaviors, or gave vocalizations only in the session. The fixed effect was whether the session was PC or MC.

We then examined factors affecting the vocal use of subjects in the PC session (Model 5; Table 1). The response variable was whether the subject gave vocalizations during affiliative interactions, and the fixed effects were degree of familiarity and rank differences between the former opponents, whether or not the original conflict included physical aggression, and occurrence of counter-aggression.

### Consequences of vocalizations

We compared the risk of receiving aggression immediately after affiliations between the former opponents by whether affiliative behaviors included vocalizations. For PC sessions, in which affiliative interactions between former opponents occurred, we compared the probability of receiving aggression after affiliative behaviors between instances in which subjects used vocalizations and those in which they did not (Model 6, Table 1). The response variable consisted of whether the subject received aggression from the former opponent from the beginning of the interaction until the end of the PC session. The fixed effects consisted of whether the original conflict included physical aggression, the degree of familiarity and rank differences between the individuals, and whether the subjects exhibited vocalizations. Whether the original conflict included counter-aggression was excluded from this analysis because a model that included this factor was not estimated accurately. We also conducted the same analysis, but replaced one fixed effect; the factor of whether vocalizations were used was replaced with the factor of whether reconciliations consisted of vocalizations accompanied by physical contact or approach, vocalizations only, or physical contact or approach only (Model 6', Table 1).

## Results

### Occurrence of affiliative interactions

Of the 300 pairs of PC-MC sessions in which aggressors were the focal subjects, affiliative behaviors occurred in 47 PC sessions with the former opponent and in 112 MC sessions with any individuals. Of the 305 sessions focusing on victims, affiliative behaviors occurred in 72 PC sessions and in 113 MC sessions. In the MC sessions, neither aggressors nor victims interacted with former opponents. The analysis revealed that in the first 1-min timeframe, the proportion of attracted PC-MC pairs (*N* = 76, mean ± SD: 12.5 ± 15.0%) exceeded those of dispersed pairs (8.3 ± 10.7%) for former opponents after the conflict (Wilcoxon signed-rank exact test: V = 257.5, *P* = 0.040). However, the proportions of dispersed pairs exceeded those of attracted pairs in the following timeframes (attracted vs. dispersed: the second, 1.9 ± 5.0% vs. 7.8 ± 10.2%, V = 563, *P* < 0.001; the third, 1.0 ± 4.1% vs. 4.1 ±8.8%, V = 156, *P* = 0.012; the fourth, 0.4 ± 2.7% vs. 3.6 ± 7.2%, V = 222.5, *P* < 0.001; the fifth, 0.1 ± 1.0% vs. 3.1 ± 6.4%, V = 171, *P* < 0.001). Thus, reconciliation occurred, but only in the first minute.

In the PC of aggressors, affiliative interactions with the former opponent occurred frequently when no counter aggression occurred and the former opponent was more familiar (generalized linear mixed models: GLMM; Model 1; Table 2). PC affiliations tended to occur between aggressors and higher-ranking opponents; however, this was not statistically significant. With regards to victims, PC affiliative interactions with the former opponent were more likely to occur when no counter-aggression occurred (Table 2). These interactions tended to occur when the original conflict included physical aggression, and when the former opponents were more familiar; however, this was not statistically significant (Table 2). Thus, the absence of counter-aggressions increased the probability of affiliation between the former opponents after conflict for both aggressors and victims; however, high familiarity with the former opponent had a significant effect only for aggressors.

**Table 2:** GLMM logistic regression results for the factors affecting the occurrence of post-conflict interactions with the former opponent.

Explanatory variables		*β* (SE)	*z*	*P*
Aggressors				
	Intercept	-1.513 (0.477)	-3.174	0.002
	Physical aggression: yes	-0.233 (0.410)	-0.567	0.571
	Counter aggression: yes	-1.284 (0.556)	-0.310	0.021
	Rank difference	0.014 (0.008)	1.867	0.062
	Familiarity	5.375 (1.683)	3.194	0.001
	The full vs. null model comparison: *N* = 300, χ^2^_4_ = 21.004, *P* < 0.0001			
Victims				
	Intercept	-0.882 (0.417)	-2.122	0.035
	Physical aggression: yes	-0.701 (0.362)	-1.935	0.059
	Counter aggression: yes	-1.419 (0.538)	-2.639	0.008
	Rank difference	-0.002 (0.007)	-0.351	0.726
	Familiarity	1.623 (2.390)	1.887	0.059
	The full vs. null model comparison: *N* = 305, χ^2^_4_ = 17.0, *P* < 0.0001			

### Risk of receiving aggression

Victims received aggression from the former opponent in 29 PC sessions, whereas aggressors received aggression from the former opponent in only four cases. We confirmed that the probability of receiving aggression is significantly higher in PC than in MC for victims (Model 2; *N* = 610, *β* ± SE = 0.713 ± 0.268, *z* = 2.660, *P* = 0.008; S1 Table). However, this was not the case for aggressors (*N* = 600, *β* ± SE = 0.226 ± 0.390, *z* = 0.581, *P* = 0.562; S1 Table). Therefore, we conducted the following examination on victims only.

**Fig 1:**
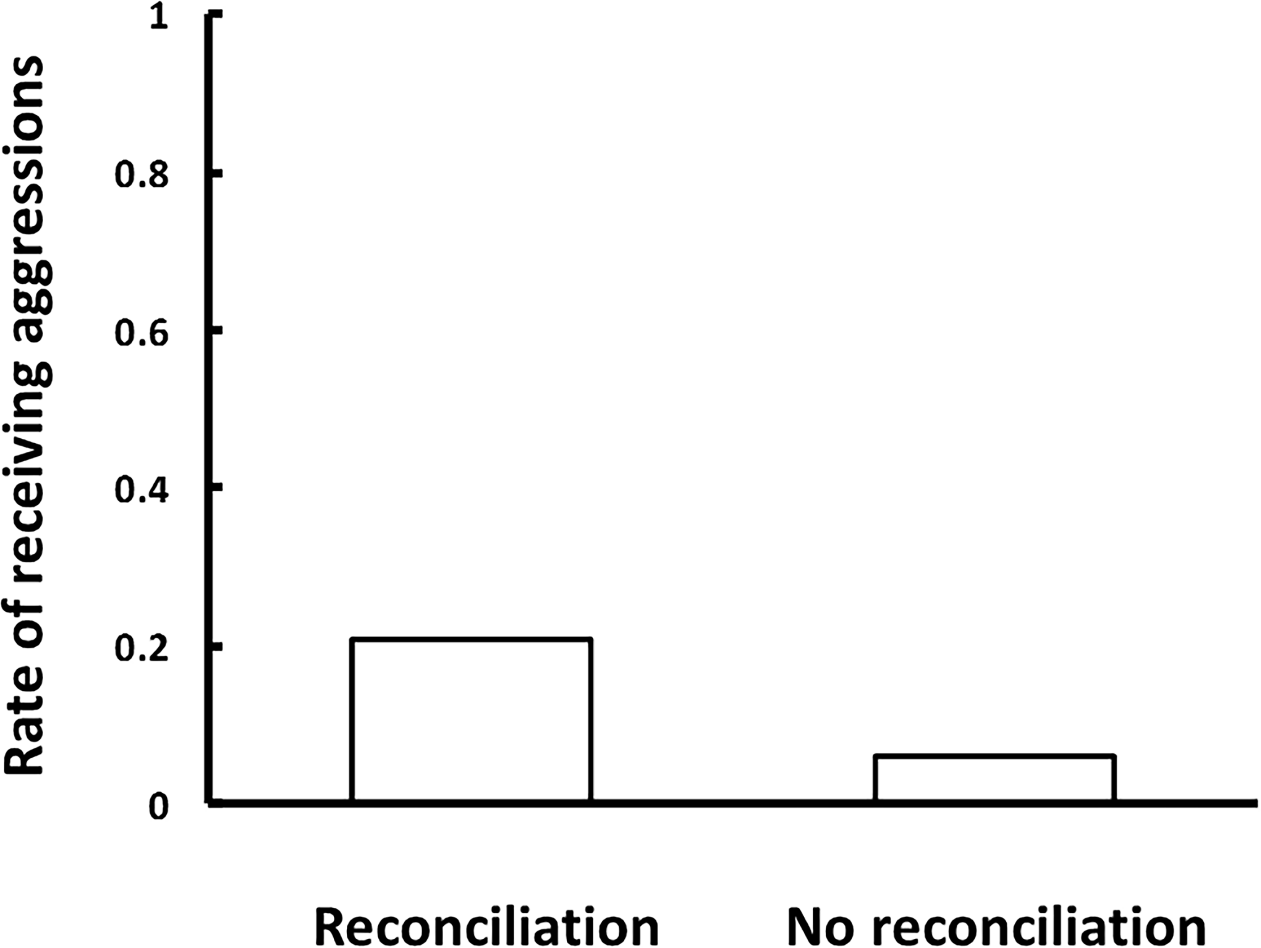
Differences between the proportions of post-conflict (PC) sessions during which victims received aggression after experiencing affiliative behaviors with the former opponent (reconciliations: *n* = 72) or not (*n* = 233).

We found that the probability of receiving aggression was higher when victims had affiliative interactions with the former opponent than when they did not (Model 3; *N* = 305, *β* ± SE = 1.339 ± 0.511, *z* = 2.622, *P* = 0.001; Fig 1, S2 Table). The characteristics of conflicts, familiarity and rank differences with the former opponent did not have significant effects. Victims were more likely to receive further aggression when affiliations with the former opponent occurred regardless the conditions.

### Vocal use after conflict

Aggressors exhibited vocalizations in 14 PC sessions (29.8%) toward opponents and in 16 MC sessions (14.3%). Victims gave vocalizations in 33 PC sessions (45.8%) toward opponents and in 10 MC sessions (9.8%). The results of the GLMM for PC and MC sessions in which affiliative interactions occurred revealed that the effect of PC or MC was significant for victims (Model 4; *N* = 185, *β* ± SE = 2.760 ± 0.557, *z* = 4.960, *P* < 0.001; S3 Table), but not for aggressors (*N* = 159, *β* ± SE = 0. 053 ± 0.465, *z* = 0.115, *P* = 0.909; S3 Table). These findings indicated that victims but not aggressors were more likely to vocalize in PC than in MC sessions. The following examinations of vocal behaviors were conducted on victims only.

**Fig 2:**
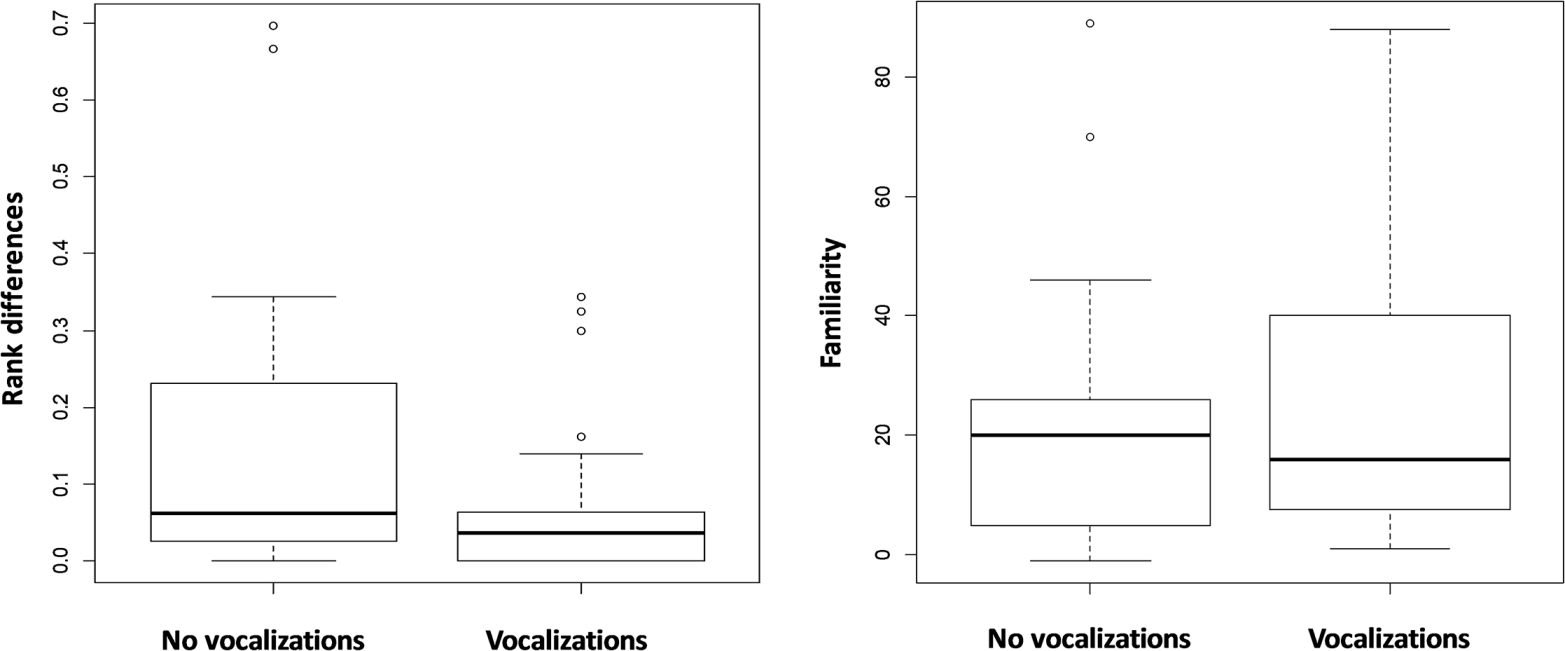
Box plots of differences in the index of familiarity (left) and rank differences (right) between opponents based on whether victims used vocalizations. Box edges represent the upper and lower quartiles, thick lines within the boxes represent medians, and open circles represent outliers (no vocalizations: *n* = 24; vocalizations: *n* = 48).

Familiarity had a significant effect on vocal use by victims among PC sessions in which affiliative interactions occurred, indicating that victims were more likely to vocalize toward less-familiar opponent (Model 5; Table 3, Fig 2). The effects of rank differences or conflict characteristics were not significant (Table 3).

**Table 3:** GLMM logistic regression results for the factors affecting vocal use of victims in post-conflict interactions with the former opponent.

Explanatory variables		*β* (SE)	*z*	*P*
	Intercept	2.021 (0.737)	2.742	0.006
	Physical aggression: yes	-0.557 (0.653)	-0.854	0.393
	Counter aggression: yes	-0.343 (1.344)	-0.255	0.799
	Rank difference	-0.008 (0.014)	-0.552	0.581
	Familiarity	-7.803 (3.076)	-2.537	0.011
	The full vs. null model comparison: *N* = 72, χ^2^_4_ = 8.72, *P* = 0.028			

### Consequences of vocalizations

**Fig 3:**
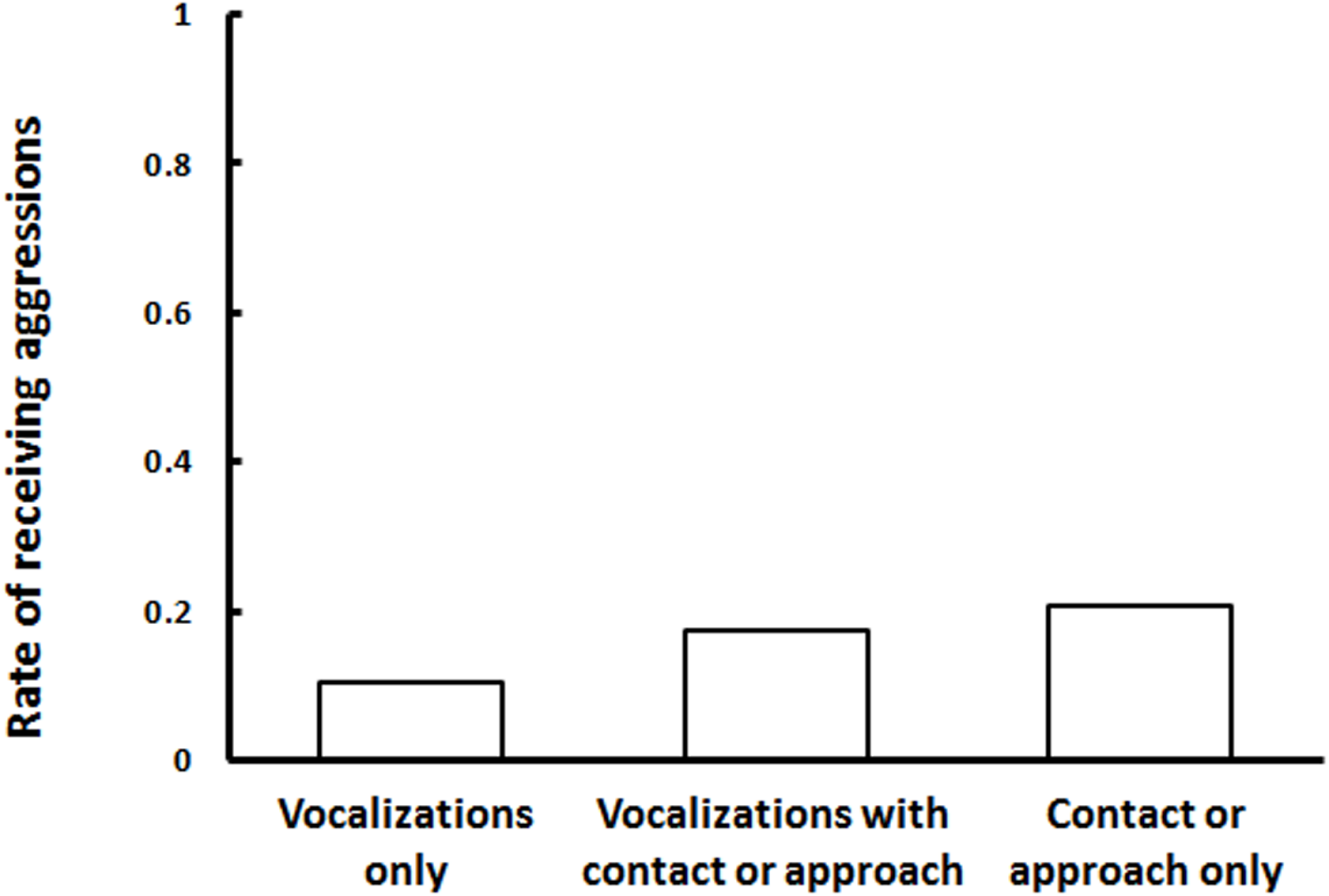
Differences between the proportion of post-conflict (PC) sessions during which victims received aggression after victims used vocalizations only (*n* = 19), vocalizations with contact or approach (*n* = 29), and contact or approach only (*n* = 24).

When victims had affiliation with the former opponent, the probability of receiving aggression did not differ between victims that exhibited vocalizations (7/48) and those that did not (Model 6; 5/24, *N* = 72, Table 4). Victims received aggression from aggressors 10.5% (2/19) of the time when they used vocalizations only; 17.2% (5/29) of the time when they both vocalized and engaged in other affiliative behaviors; and 20.8% (5/24) of the time when they used affiliative behaviors other than vocalizations. We also compared probabilities based on whether affiliative behaviors consisted of vocalizations with physical contact or approach, vocalizations only, or physical contact or approach only; however, no significant differences were detected among these three types of affiliative behaviors (Model 6'; *N* = 72, χ^2^_2_ = 1.016, *P* = 0.602, Table 5, Fig 3). Thus, risk of further aggression did not decrease significantly after vocal affiliative interactions.

**Table 4:** GLMM logistic regression results for the effect of vocal use, conflict characteristics, and relationship with the former opponent on the probability of receiving further aggression after post-conflict interactions.

Explanatory variables	*β* (SE)	*z*	*p*
Intercept	-1.014 (1.244)	-0.815	0.415
Physical aggression: yes	1.283 (0.768)	1.672	0.095
Rank difference	-0.006 (0.020)	-0.292	0.770
Familiarity	3.482 (3.582)	0.972	0.331
Victims used vocalization: yes	-0.528 (0.853)	-0.618	0.536
The full vs. null model comparison: *N* = 72, χ^2^_4_ = 8.05, *P* = 0.048			

**Table 5:** GLMM logistic regression results for the effect of type of interaction, conflict characteristics, and relationship with the former opponent on the probability of receiving further aggression after post-conflict interactions.

Explanatory variables	*β* (SE)	*z*	*p*
Intercept	-1.756 (1.202)	-1.462	0.144
Physical aggression: yes	1.222 (0.728)	1.679	0.093
Rank difference	-0.005 (0.019)	-0.268	0.789
Familiarity	2.662 (2.354)	1.131	0.258
Type of behavior: vocalization only	-1.201 (1.247)	-0.963	0.336
Type of behavior: vocalization with approach or contact	-0.294 (0.810)	-0.363	0.717
The full vs. null model comparison: *N* = 72, χ^2^_5_ = 8.458, *P* = 0.036			

## Discussion

We examined the use of vocalizations and their possible role in post-conflict situations for victims of aggression in Japanese macaques. Reconciliation was observed to occur in the first minute after conflict. As reported in previous studies on Barbary macaques (*Macaca sylvanus* [37, 38], victims in our study were more likely to receive further aggression when they had affiliative interaction with the former aggressor than when no interaction occurred. Aggressors were more likely to have interactions with former opponents with whom they were more familiar, whereas the effect of familiarity was not statistically significant for victims. When compared to other primates, Japanese macaques frequently engage with more intense aggression [26, 39] than Celebes crested macaques (*Macaca nigra* [40] do. In general, the occurrence of reconciliation is more frequent in Tonkean macaques than in rhesus or Japanese macaques [41, 42]. Among the Japanese macaques in our study group, the proportion of attracted pairs was 12.5%, which falls within the range previously reported for Japanese and rhesus macaques [42, 43]; however, conciliatory tendency was relatively low in the Arashiyama group (4.2%) when compared to observation made in an earlier study (8.4%) [11], suggesting that victims tended to avoid former aggressors in post-conflict situations, even if the aggressors were familiar to them. Both aggressors and victims were less likely to exhibit reconciliation when counter-aggression occurred, indicating that conflicts often remained unsettled in these cases.

When affiliative interactions with the former opponent occurred, victims emitted vocalizations more frequently in PC than in MC sessions, and were more likely to use vocalizations when the former opponents were less familiar, as we predicted. Previous studies on hamadryas baboons and mandrills showed that non-contact behaviors were often used toward inaccessible individuals [13, 14]. Victims were supposed to use vocalizations when they interacted with less predictable individuals in the same way as other non-contact behaviors, because vocalizations can be used at a distance from the former opponent. As for aggressors, vocal use did not increase in PC. Previous research on baboons has been mostly focused on vocal use by high-ranking individuals in post-conflict situations [19, 24], in contrast, our findings suggest that victims, not aggressors, were more likely to use vocalizations in post-conflict situations. Tonkean macaques exhibit a wide variety of forms of reconciliation, including facial expressions, lip-smacking, clasping, and mounting, whereas rhesus macaques primarily used grooming [42]. Our study suggests that despotic Japanese macaques also use behaviors other than grooming during reconciliation; however, vocalization was used at a distance, in contrast to the behaviors exhibited by tolerant macaque species. This difference in the types of behaviors exhibited by different species is probably due to the despotic structure of Japanese macaque societies. Further studies on a broader range of species of macaques are required to clarify whether they exhibit similar distance behaviors during post-conflict situations as has been seen in other despotic macaque societies.

We predicted that vocalizations would be used more frequently during interactions with higher-ranking opponents; however, no significant effects of rank differences were detected. Almost all victims were subordinate to aggressors in the present study; therefore, rank differences between victims and opponents appeared to have less of an effect than general dominant or subordinate relationships.

A study on mandrills revealed that both contact and non-contact interactions are effective in preventing further aggression [14]; here, we predicted that vocal behaviors reduce the risk of further aggression compared with contact or approach without vocalizations, because victims can vocalize at a distance from former aggressors. However, the probability of receiving aggression was not significantly lower after victims used vocalizations only or used vocalizations in combination with approach or contact, than approach or contact only. Grunts, girneys, and coos are honest signals that callers use to indicate no hostile intent [17, 18]. Our findings suggest that even when victims conveyed non-hostile intent from a distance, they were not able to reduce hostility of aggressors or avoid further aggression. This is possibly because the probability of further aggression was, to some extent, unavoidable in this macaque group. However, differences in the type of interactions may go undetected due to the small sample size. Therefore, additional future research is required to better understand the function of vocalizations.

In summary, female Japanese macaques interacted with other individuals in post-conflict situations using different forms of behavior depending on the degree of uncertainty. Our study revealed that victims utilized vocal behaviors towards less predictable former opponent in post-conflict situations; however, benefit of vocalization use by victims following conflicts remain unclear. The risk of receiving aggression was not significantly reduced even after vocalizations were used. It has been shown that victims reduce the probability of being the target of further aggression by redirecting aggression toward third-party individuals [44]; thus, victims can choose not to interact with the former opponent or to engage in redirect aggression. Victims may engage in affiliation with the former opponent because signaling non-hostile intent plays an important role in the long-term maintenance of non-agonistic relationships, although it may also entail some immediate risk. A previous study of the same group of Japanese macaques that focused on the long-term effects of affiliation, including vocal behaviors in post-conflict situations, concluded that when no affiliative interactions between former opponents occurred after a conflict, the affiliation rates were lower, and aggression rates were higher over the subsequent 10 days compared to baseline levels [23]. Vocalizations can be used from a distance to signal that victims intend to cease conflict with the former opponent. Victims may have used vocalizations when the former opponent was inaccessible individuals, but when it is detrimental to leave a conflict unsettled. Further research is required to clarify whether functions associated with the repair of bonds and reductions in further aggression differ when vocalizations or other forms of behaviors are expressed.

## Supporting information

S1 Table GLMM logistic regression results for the effect of situation on whether the subjects received aggression. (DOCX)

S2 Table GLMM logistic regression results for the factors affecting the probability of receiving post-conflict aggression in victims. (DOCX)

S3 Table GLMM logistic regression results for the effect of situation on whether the subjects used vocalizations in interacting with former opponents. (DOCX)

S4 Table Dataset. (XLSX)
