# Supplementary material for: Vocalizations during post-conflict affiliations from victims toward aggressors based on uncertainty in Japanese macaques
Source: PLoS One. 2017 May 30;12(5):e0178655. doi: 10.1371/journal.pone.0178655 (PMC5448802; doi:10.1371/journal.pone.0178655)
Supplement: S1 Table — (DOCX) [file pone.0178655.s001.docx]

| S1. Table GLMM logistic regression results for the effect of situation on whether the subjects received aggression. | | | | |
| --- | --- | --- | --- | --- |
| Explanatory variables | | *β* (SE) | *z* | *p* |
| Aggressor | |  |  |  |
|  | Intercept | -3.461 (0.422) | -8.208 | < 0.0001 |
|  | PC or MC: PC | 0.226 (0.390) | 0.581 | 0.562 |
| The full vs. null model comparison: *N* = 600, χ^2^_1_ = 18.887, *P* < 0.0001 | | | | |
| Victim | |  |  |  |
|  | Intercept | -2.629 (0.267) | -9.837 | < 0.0001 |
|  | PC or MC: PC | 0.713 (0.268) | 2.660 | 0.008 |
| The full vs. null model comparison: *N* = 610, χ^2^_1_ = 36.176, *P* < 0.0001 | | | | |
