# Supplementary material for: Vocalizations during post-conflict affiliations from victims toward aggressors based on uncertainty in Japanese macaques
Source: PLoS One. 2017 May 30;12(5):e0178655. doi: 10.1371/journal.pone.0178655 (PMC5448802; doi:10.1371/journal.pone.0178655)
Supplement: S2 Table — (DOCX) [file pone.0178655.s002.docx]

| S2. Table GLMM logistic regression results for the factors affecting the probability of receiving post-conflict aggression in victims. | | | |
| --- | --- | --- | --- |
| Explanatory variables | *β* (SE) | *z* | *p* |
| Intercept | -2.773 (0.610) | -4.549 | < 0.0001 |
| Physical aggression: yes | -0.813 (0.511) | -1.592 | 0.111 |
| Counter aggression: yes | -1.213 (1.067) | -1.137 | 0.256 |
| Rank difference | 0.005 (0.010) | 0.461 | 0.645 |
| Familiarity | 1.935 (1.628) | 1.189 | 0.235 |
| Affiliation with former opponents: yes | 1.339 (0.511) | 2.622 | 0.001 |
| The full vs. null model comparison: *N* = 305, χ^2^_5_ = 14.701, *P* = 0.005 | | | |
