# Supplementary material for: Vocalizations during post-conflict affiliations from victims toward aggressors based on uncertainty in Japanese macaques
Source: PLoS One. 2017 May 30;12(5):e0178655. doi: 10.1371/journal.pone.0178655 (PMC5448802; doi:10.1371/journal.pone.0178655)
Supplement: S3 Table — (DOCX) [file pone.0178655.s003.docx]

| S3. Table GLMM logistic regression results for the effect of situation on whether the subjects used vocalizations in interacting with former opponents. | | | | |
| --- | --- | --- | --- | --- |
| Explanatory variables | | *β* (SE) | *z* | *p* |
| Aggressor | |  |  |  |
|  | Intercept | -1.518 (0.344) | -4.407 | < 0.0001 |
|  | PC or MC: PC | 0.053 (0.465) | 0.115 | 0.909 |
| The full vs. null model comparison: *N* = 159, χ^2^_1_ = 38.8, *P* < 0.0001 | | | | |
| Victim | |  |  |  |
|  | Intercept | -2.047 (0.459) | -4.463 | < 0.0001 |
|  | PC or MC: PC | 2.760 (0.557) | 4.960 | < 0.0001 |
| The full vs. null model comparison: *N* = 185, χ^2^_1_ = 25.2, *P* < 0.0001 | | | | |
